# ‘Keep an open mind’: using qualitative research to make recruitment easier in the by-band randomised controlled trial

**DOI:** 10.1186/1745-6215-14-S1-O54

**Published:** 2013-11-29

**Authors:** Sangeetha Paramasivan, Chris Rogers, Graziella Mazza, Jane Blazeby, Jenny Donovan

**Affiliations:** 1 School of Social and Community Medicine, University of Bristol, Bristol, UK; 2 School of Clinical Sciences, Clinical Trials and Evaluation Unit, University of Bristol, Bristol, UK

## Background

Surgical randomised controlled trials (RCTs) are acknowledged to be particularly challenging to conduct and recruit to. Within a programme of research to understand and optimise recruitment to RCTs, we undertook qualitative research in the By-Band RCT (comparison of gastric bypass and gastric band operations for morbid obesity).

## Methods

In Phase I, challenges to recruitment were explored through thematic analysis of a) interviews with trial staff and recruiters and b) audio-recordings and observations of recruitment appointments. In Phase II, anonymised findings presented to recruiters in group and individual feedback sessions led to changes in information provision and logistical aspects of recruitment.

## Results

In Phase I, interview data revealed recruiters’ varying levels of equipoise and preferences, and patient pathway charting identified complex clinical arrangements. Audio-recordings and observations of recruitment appointments highlighted five key challenges to recruitment: a) there was little discussion of By-Band b) By-Band was not well integrated into the clinical service c) eligibility assessment was unclear d) patient preferences were accepted at face value and e) information provision was not tailored to the patient. In Phase II, suggestions to recruiters included integrating the RCT within current service provision, streamlining eligibility assessment, exploring patient preferences, providing balanced and tailored information, and requesting patients to keep an open mind throughout the appointment. Recruitment rates increased from 4% prior to feedback to 30% and 64% in the two months following feedback. Research is on-going to further optimise recruitment.

## Conclusion

Qualitative research methods are well placed to optimise recruitment rates in surgical RCTs.

